# Roe and Metz identical-test simulation model for validating multi-reader methods of analysis for comparing different radiologic imaging modalities

**DOI:** 10.1117/1.JMI.10.S1.S11916

**Published:** 2023-07-05

**Authors:** Stephen L. Hillis

**Affiliations:** University of Iowa, Departments of Radiology and Biostatistics, Iowa City, Iowa, United States

**Keywords:** receiver operating characteristic curve, diagnostic radiology, Roe and Metz, Obuchowski and Rockette, Gallas, type I error, multi-reader multi-case, multi-reader multi-case analysis

## Abstract

**Purpose:**

The most frequently used model for simulating multi-reader multi-case (MRMC) data that emulate confidence-of-disease ratings from diagnostic imaging studies has been the Roe and Metz model, proposed by Roe and Metz in 1997 and later generalized by Hillis (2012), Abbey et al. (2013), and Gallas and Hillis (2014). These models have been used for evaluating MRMC analysis and sample size methods. The models suggested in these papers for assessing type I error have been null models, where the expected area under the receiver-operating-characteristic curve across readers is the same for each test. However, for these null models, there are other differences that would not exist if the two tests were identical. None of the papers mentioned above discuss how to formulate a null model that is also an identical-test model, where the two tests are identical in all respects. The purpose of this paper is to show how to formulate a Roe and Metz identical-test model and to show its usefulness for validating the error covariance constraints employed by the Obuchowski-Rockette (1995) method.

**Approach:**

For a given Roe-and-Metz model, the corresponding Roe-and-Metz identical-test model is derived by modifying the Roe and Metz null model under the assumption that the two tests are identical.

**Results:**

The importance of the Obuchowski-Rockette model constraints for avoiding negative variance estimates is established using data simulated from the Roe and Metz identical-test model. It is also shown that negative variance estimates can occur at nontrivial rates when the two tests are not identical but somewhat “close” to being identical.

**Conclusions:**

The findings of this paper are important because it has recently been shown (Hillis, 2022) that the commonly used MRMC method proposed by Gallas (2006) and Gallas et al. (2009) uses the same test statistic as the unconstrained Obuchowski-Rockette method.

## 1 Introduction

For the typical diagnostic radiology study, several readers (usually 4 to 10 radiologists) assign confidence-of-disease ratings to each case (i.e., subject) based on one or more corresponding radiologic images, using one or more tests (typically imaging modalities), with the numbers of diseased and nondiseased cases each typically between 25 and 100. The resulting data are called multi-reader multi-case (MRMC) data. These studies are typically used to compare different imaging modalities with respect to reader performance. Often measures of reader performance are functions of the estimated receiver-operating-characteristic (ROC) curve, such as the area under the ROC curve (AUC). Throughout we assume AUC is the reader performance metric of interest. Two commonly used methods for analyzing reader performance outcomes that allows conclusions to generalize to both the reader and case populations are the method proposed by Obuchowski and Rockette^1^ and later modified by Hillis,^2^ which will be referred to as the “OR” method, and the method proposed by Gallas^3^ and Gallas et al.,^4^ which will be referred to as the “Gallas” method.

The most frequently used model for simulating MRMC data has been the model first proposed by Roe and Metz^5^ and later generalized by Hillis,^6^ Abbey^7^ and Gallas and Hillis.^8^ We will refer to each of these models as a “Roe and Metz” or “RM” model when there is no need to distinguish between them. These RM models have been used for evaluating MRMC analysis and sample size methods. As discussed by Hillis,^9^ these RM models generate continuous confidence-of-disease ratings based on an underlying binormal model for each reader, with the separation between the normal and abnormal rating distributions varying across readers.

The parameter settings included in the original RM paper^5^ result in RM “null” models, where the mean AUC across readers is the same for each test. These null models are useful for evaluating the performance of MRMC methods with respect to type I error for the hypothesis of equal test AUCs. However, these null models can result in correlations for the simulated ratings that would be different if the two tests were identical. For example, it will be shown that between-test correlations of case ratings generated from the RM null model are less than or equal to corresponding correlations when the two tests are identical.

An RM null model where the two tests are identical will be referred to as an “identical-test” model. Although there is no reason to compare two tests that are known to be identical, sometimes it is of interest to compare two tests that are quite similar is most respects, e.g., when the two tests are the same imaging modality but used with slightly different radiation doses. For this situation a researcher likely would want to test if the lower-dose modality is noninferior or equivalent to the higher dose modality. For such situations, it is important to know that the MRMC analysis method being used performs well when the tests are close to being identical, not only in terms of AUC, but in other ways.

A discussion of how to determine parameter settings that result in an identical-test model is not provided in the original RM paper or in any of the previously mentioned papers that generalize the original RM model. The purpose of this paper is to show how to formulate an RM identical-test model and to show its usefulness for validating the need for the error covariance constraints employed by the OR method. A summary of the paper is as follows: a review of the various RM models is provided in Sec. 2, the definition and derivation of an RM identical-test model are provided in Sec. 3 with illustrative examples in Sec. 4, a brief review of the conventional OR, unconstrained OR and Gallas methods is provided in Sec. 5 with simulation studies comparing the methods in Sec. 6, a discussion of how a negative OR variance can occur is presented in Sec. 7 with illustrative simulation studies in Sec. 8, followed by a summary and discussion in Sec. 9.

## 2 Roe and Metz null Models: Original, Constrained, and Unconstrained Unequal-Variance

### 2.1 Original RM Null Model

Let X denote a confidence-of-disease rating assigned by a reader to a case; X is often called a decision variable (DV). The original RM simulation model proposed by Roe and Metz^5^ is a mixed four-factor (test, reader, case, and truth) ANOVA model for X with case nested within truth; test, reader, and truth crossed; test and truth treated as fixed factors; and reader and case treated as random factors.

Using their notation, their null model is given as

$$X_{ijkt}=\mu_{\mathrm{pos}}I_{\left\{t=+\right\}}+R_{jt}+C_{kt}+(\tau R{)}_{ijt}+(\tau C{)}_{ikt}+(RC{)}_{jkt}+(\tau RC{)}_{ijkt}+E_{ijkt},$$ (1)

where $X_{ijkt}$ denotes the confidence-of-disease rating assigned to case k of truth state t by reader j when reading under test i, with t = “−” indicating a nondiseased case and t = “+” indicating a diseased case. Here $\mu_{\mathrm{pos}}$ is the expected difference in the means for the diseased and nondiseased DV distributions, $I_{\left\{t=+\right\}}$ is an indicator function that takes the value 1 when t=+ and 0 when t=−, $R_{jt}$ is the interaction effect of reader j and truth state t, $C_{kt}$ is the effect of case k nested within truth state t, the multiple symbols in parentheses denote interactions, and $E_{ijkt}$ is the error term. By comparison, the nonnull model given by Roe and Metz is the same as Eq. (1) except that it also includes a test-by-truth interaction term, denoted by $\tau_{it}$, which is implicitly set to zero in the null model Eq. (1).

All effects in Eq. (1) are random except for $\mu_{\mathrm{pos}}$. The random effects are mutually independent and normally distributed with zero means. Roe and Metz denote the corresponding variance components by $\sigma_{R}^{2}$, $\sigma_{C}^{2}$, $\sigma_{\tau R}^{2}$, $\sigma_{\tau C}^{2}$, $\sigma_{RC}^{2}$, $\sigma_{\tau RC}^{2}$, and $\sigma_{E}^{2}$. They note that $\sigma_{\tau RC}^{2}$ and $\sigma_{E}^{2}$ cannot be estimated separately for this model with no replications, and hence define

$$\sigma_{\varepsilon }^{2}\equiv \sigma_{\tau RC}^{2}+\sigma_{E}^{2}.$$ (2)

Although not mentioned by Roe and Metz, the omission of effects that do not depend on truth is justified by the invariance of the ROC curve to location shifts; thus, inclusion of these terms would not change the ROC curve for a given reader. Note that interactions with truth are denoted only by a t subscript in Eq. (1). Roe and Metz constrain the sum of the error variance and variance components involving case to be equal to one:

$$\sigma_{C}^{2}+\sigma_{\tau C}^{2}+\sigma_{RC}^{2}+\sigma_{\varepsilon }^{2}=1.$$ (3)

It can be shown (e.g., Hillis^9^) that the reader nondiseased and diseased DV distributions have unit variances (and hence their ROC curves are symmetric about the negative 45 deg diagonal), with the reader true AUCs varying across the reader population and having the same expectation for each test. Furthermore, a randomly selected reader has the same ROC curve under each test.

### 2.2 Constrained and Unconstrained Unequal-Variance RM Null Models

In practice, estimated binormal-model nondiseased and diseased DV variances for a fixed reader are often different, with diseased subjects typically having more variable case ratings. To better emulate real data, Hillis^6^ modified the original RM model by allowing the error variance and variance components involving case to depend on truth, with variance components involving diseased cases set equal to those involving normal cases multiplied by the factor $1/b^{2}$, b>0. Specifically, the null model is given by Eq. (1) with variance components (using an obvious notation) denoted as

$$\sigma_{R}^{2},\sigma_{\tau R}^{2},\sigma_{C(-)}^{2},\sigma_{\tau C(-)}^{2},\sigma_{RC(-)}^{2},\sigma_{\varepsilon (-)}^{2},\sigma_{C(+)}^{2},\sigma_{\tau C(+)}^{2},\sigma_{RC(+)}^{2},\sigma_{\varepsilon (+)}^{2},$$ (4)

with

$$\sigma_{C(+)}^{2}=b^{-2}\sigma_{C(-)}^{2},\sigma_{\tau C(+)}^{2}=b^{-2}\sigma_{\tau C(-)}^{2},\sigma_{RC(+)}^{2}=b^{-2}\sigma_{RC(-)}^{2},\sigma_{\varepsilon (+)}^{2}=b^{-2}\sigma_{\varepsilon (-)}^{2}.$$ (5)

Similar to Eq. (3), the constraint

$$\sigma_{C(-)}^{2}+\sigma_{\tau C(-)}^{2}+\sigma_{RC(-)}^{2}+\sigma_{\varepsilon (-)}^{2}=1,$$ (6)

is imposed. It follows that

$$\sigma_{C(+)}^{2}+\sigma_{\tau C(+)}^{2}+\sigma_{RC(+)}^{2}+\sigma_{\varepsilon (+)}^{2}=b^{-2}.$$

Following Hillis,^6^ we refer to this as the “constrained unequal-variance RM null model.” It follows^6^ from Eq. (5) that setting b=1 results in the original RM model and that b is the conventional binormal-model slope coefficient for each reader’s ROC curve.

A more general RM null model, called the “unconstrained unequal-variance RM null model” by Hillis,^9^ results if the variance components $\sigma_{C(+)}^{2}$, $\sigma_{\tau C(+)}^{2}$, $\sigma_{RC(+)}^{2}$, and $\sigma_{\varepsilon (+)}^{2}$ are not constrained to satisfy any particular relationship with $\sigma_{C(-)}^{2}$, $\sigma_{\tau C(-)}^{2}$, $\sigma_{RC(-)}^{2}$, and $\sigma_{\varepsilon (-)}^{2}$. This model includes the original and constrained unequal-variance RM null models as special applications.

### 2.3 Comparison of the RM Null Models

The original RM null model and the constrained and unconstrained unequal-variance RM null models all have the same mixed linear model formulation, given by Eq. (1); all of them also constrain the sum of the variance components corresponding to effects involving nondiseased cases to be equal to 1, as given by Eq. (6). The null models differ only with respect to their constraints on the variance components corresponding to effects involving diseased cases, with the original RM model requiring that the variance components be the same as those for the nondiseased cases, the constrained unequal-variance model requiring that they differ by a factor of $1/b^{2}$ from those for the nondiseased cases, and the unconstrained unequal-variance model not placing any constraints on them.

## 3 Proposed RM Identical-Test Model

### 3.1 Definition of Identical-Test Model

I define two tests to be “identical” if they are the same in all respects. I will derive an RM identical-test model by applying this definition to an unconstrained unequal-variance RM null model; since this model includes the original and constrained unequal-variance RM null models as specific applications, the derivation can also be applied to those models. Recall that the unconstrained unequal-variance RM null model is defined by mixed linear model Eq. (1) with variance components given by Eq. (4) subject only to constraint Eq. (6).

### 3.2 Derivation of an RM Identical-Test Model

In this section I derive the RM identical-test model by modifying the unconstrained unequal-variance RM null model. The definition of identical tests implies that model effects (excluding the error term) cannot differ by test in an RM identical-test model. Thus, if tests i=1 and i=2 are identical, it follows that model effects in Eq. (1) that include test do not depend on the value of the test subscript i. Specifically, ${\left(\tau R\right)}_{ijt}$, $(\tau C{)}_{ikt}$, and ${\left(\tau RC\right)}_{ijkt}$ in Eq. (1) cannot depend on the value of subscript i; hence $(\tau R{)}_{1jt}=(\tau R{)}_{2jt}$, $(\tau C{)}_{1kt}=(\tau C{)}_{2kt}$, and $(\tau RC{)}_{1jkt}=(\tau RC{)}_{2jkt}$.

Thus I can derive the RM identical-test model from the unequal-variance RM null model using the following result.

**Result 1.** Setting test subscript values (*i*) in Eq. (1) equal to 1 for model effects (excluding the error term) results in the corresponding RM identical-test model.

Applying Result 1 results in none of the model effects that include test depending on the value of the test subscript, since it will be the same for all of these effects.

Applying Result 1 to the unequal-variance RM null model given by mixed linear model Eq. (1) with variance components Eq. (4) subject only to constraint Eq. (6) results in the identical-test RM null model

$${\overset{˜}{X}}_{ijkt}=\mu_{\mathrm{pos}}I_{\left\{t=+\right\}}+R_{jt}+C_{kt}+(\tau R{)}_{1jt}+(\tau C{)}_{1kt}+(RC{)}_{jkt}+(\tau RC{)}_{1jkt}+E_{ijkt},$$

where ${\overset{˜}{X}}_{ijkt}$ is the identical-test model DV. Consolidating random effects results in the equivalent model

$${\overset{˜}{X}}_{ijkt}=\mu_{\mathrm{pos}}I_{\left\{t=+\right\}}+{\overset{˜}{R}}_{jt}+{\overset{˜}{C}}_{kt}+(\overset{˜}{RC}{)}_{jkt}+{\overset{˜}{E}}_{ijkt},$$ (7)

where

$${\overset{˜}{R}}_{jt}=R_{jt}+(\tau R{)}_{1jt},\;{\overset{˜}{C}}_{kt}=C_{kt}+(\tau C{)}_{1kt},\;(\overset{˜}{RC}{)}_{jkt}=(RC{)}_{jkt}+(\tau RC{)}_{1jkt},\;{\overset{˜}{E}}_{ijkt}=E_{ijkt}.$$

Corresponding variance components for ${\overset{˜}{R}}_{jt}$, ${\overset{˜}{C}}_{kt}$, $(\tilde{RC}{)}_{jkt}$, and ${\overset{˜}{E}}_{ijkt}$ are given as

$$\sigma_{\overset{˜}{R}}^{2}=\sigma_{R}^{2}+\sigma_{\tau R}^{2},$$ (8)

$$\sigma_{\overset{˜}{C}(t)}^{2}=\sigma_{C(t)}^{2}+\sigma_{\tau C(t)}^{2},$$ (9)

$$\sigma_{\tilde{RC}(t)}^{2}=\sigma_{RC(t)}^{2}+\sigma_{\tau RC(t)}^{2},$$ (10)

$$\sigma_{\overset{˜}{E}(t)}^{2}=\sigma_{\varepsilon (t)}^{2}-\sigma_{\tau RC(t)}^{2}.$$ (11)

It follows from Eqs. (6) and (9)–(11) that

$$\sigma_{\overset{˜}{C}(-)}^{2}+\sigma_{\overset{˜}{RC}(-)}^{2}+\sigma_{\overset{˜}{E}(-)}^{2}=1.$$ (12)

In summary, the RM identical-test model derived from the unconstrained unequal-variance null RM model is given by model Eq. (7) with variance components Eqs. (8)–(11) and constraint Eq. (12).

Because the original, constrained unequal variance, and unconstrained unequal variance RM null models specify values for $\sigma_{\varepsilon (t)}^{2}=\sigma_{\tau RC(t)}^{2}+\sigma_{E(t)}^{2}$ without specifying specific values for either $\sigma_{E(t)}^{2}$ or $\sigma_{\tau RC(t)}^{2}$, values must be assigned to $\sigma_{\tau RC(t)}^{2}$, t=+,− for the null model in order to determine values for $\sigma_{\tilde{RC}(t)}^{2}$ and $\sigma_{\overset{˜}{E}(t)}^{2}$ in the identical-test model using Eqs. (10) and (11).

For simplicity, for the remainder of this paper I will assume

$$\sigma_{\tau RC(-)}^{2}=\sigma_{\tau RC(+)}^{2}=0$$ (13)

in the unconstrained unequal variance RM model, resulting in

$$\sigma_{\tilde{RC}(t)}^{2}=\sigma_{RC(t)}^{2},$$ (14)

$$\sigma_{\overset{˜}{E}(t)}^{2}=\sigma_{\varepsilon (t)}^{2},$$ (15)

in the identical-test model. On the other hand, if the values for $\sigma_{\tau RC(-)}^{2}$ or $\sigma_{\tau RC(+)}^{2}$ are specified, then the values for $\sigma_{\tilde{RC}(t)}^{2}$ and $\sigma_{\overset{˜}{E}(t)}^{2}$ can be computed using Eqs. (10) and (11).

When using the identical-test model for simulations, ratings $X_{1jkt}$ and $X_{2jkt}$ are simulated, corresponding to tests 1 and 2, respectively. But since the only term on the right of Eq. (7) that depends on test is the error term, it follows that for a given reader, case, and truth status, the ratings for the two tests will differ only because their error term values will not be the same.

Note that because the derivation was based on an RM null model, the resulting RM identical-test model is also an RM null model and is a specific application of the unconstrained unequal-variance RM null model.

### 3.3 Comparison of the RM Null Model and the Corresponding RM Identical-Test Model

The following relationships for the ratings generating from an unconstrained unequal-variance RM null model and its corresponding RM identical-test model Eqs. (7)–(15) can be shown.

1. Conditional on disease status, the RM null model and the corresponding RM identical-test model result in the same rating distributions for both tests. Specifically, for either test 1 (i=1) or test 2 (i=2), $X_{ijk-}$ and ${\overset{˜}{X}}_{ijk-}$ have $N(0,\sigma_{R}^{2}+\sigma_{\tau R}^{2}+1)$ distributions and $X_{2jk+}$ and ${\overset{˜}{X}}_{1jk+}$ have $N(0,\sigma_{R}^{2}+\sigma_{\tau R}^{2}+\sigma_{C(+)}^{2}+\sigma_{\tau C(+)}^{2}+\sigma_{RC(+)}^{2}+\sigma_{\varepsilon (+)}^{2})$ distributions, where “$N(0,\sigma^{2})$” indicates a normal distribution with mean 0 and variance $\sigma^{2}$.
2. Within-test rating covariances are the same for both models. Specifically, for either test 1 (i=1) or test 2 (i=2), $\mathrm{cov}(X_{ijkt},X_{ij^{\prime }k^{\prime }t^{\prime }})=\mathrm{cov}({\overset{˜}{X}}_{ijkt},{\overset{˜}{X}}_{ij^{\prime }k^{\prime }t^{\prime }})$. (Note: here and for relationship 3 below we do not assume $j\neq j^{\prime },k\neq k^{\prime }$ or $t\neq t^{\prime }$.) For example, the covariance between ratings for two different nondiseased cases for the same reader and test is given by $\underset{k^{\prime }\neq k}{\mathrm{cov}}(X_{ijk-},X_{ijk^{\prime }-})=\sigma_{R}^{2}+\sigma_{\tau R}^{2}=\sigma_{\overset{˜}{R}}^{2}=\underset{k^{\prime }\neq k}{\mathrm{cov}}({\overset{˜}{X}}_{ijkt},{\overset{˜}{X}}_{ijk^{\prime }t})$, and hence is the same for both models.
3. For the RM null model, between-test covariances are the same or less than corresponding RM identical-test model between-test covariances. That is, $\mathrm{cov}(X_{1jkt},X_{2j^{\prime }k^{\prime }t^{\prime }})\leq \mathrm{cov}({\overset{˜}{X}}_{1jkt},{\overset{˜}{X}}_{1j^{\prime }k^{\prime }t^{\prime }}).$ For example, the covariance between ratings for two different nondiseased cases for the same reader but for different tests is given as $\underset{k^{\prime }\neq k}{\mathrm{cov}}(X_{1jk-},X_{2jk^{\prime }-})=\sigma_{R}^{2}\leq \sigma_{R}^{2}+\sigma_{\tau R}^{2}=\sigma_{\overset{˜}{R}}^{2}=\underset{k^{\prime }\neq k}{\mathrm{cov}}({\overset{˜}{X}}_{1jk-},{\overset{˜}{X}}_{2jk^{\prime }-})$.

In summary, we see that the only difference between the rating distributions for the two models is that the between-test covariances for the unconstrained unequal-variance RM model can be less than those for the RM identical-test model.

### 3.4 RM Identical-Test Model Expressed in Terms of a Null Unconstrained Unequal-Variance RM Model with Altered Variance Components

It follows from Eqs. (7)–(15) that the RM identical-test model can be expressed in terms of an unconstrained unequal-variance RM null model, with RM identical-test variance components (indicated by an overline) defined in terms of the unconstrained unequal-variance RM null model variance components as follows, with t=−,+

$$\overset{¯}{\sigma_{R}^{2}}=\sigma_{R}^{2}+\sigma_{\tau R}^{2},$$ (16)

$$\bar{\sigma_{\tau R}^{2}}=0,$$ (17)

$$\bar{\sigma_{C(t)}^{2}}=\sigma_{C(t)}^{2}+\sigma_{\tau C(t)}^{2},$$ (18)

$$\bar{\sigma_{\tau C(t)}^{2}}=0,$$ (19)

$$\bar{\sigma_{RC(t)}^{2}}=\sigma_{RC(t)}^{2},$$ (20)

$$\bar{\sigma_{\tau RC(t)}^{2}}=0,$$ (21)

$$\bar{\sigma_{E(t)}^{2}}=\sigma_{\varepsilon (t)}^{2}.$$ (22)

The advantage of this approach is that for simulations, an unconstrained unequal-variance RM null model that is already programmed can be easily modified to produce identical-test simulations by altering the values of the null model variance components using Eqs. (16)–(22).

### 3.5 General Definition of an RM Identical-Test Model

It follows from Eqs. (12) and (16)–(22) that an unconstrained unequal-variance null RM is an RM identical-test model if it can be expressed by mixed linear model Eq. (1) with

$$\sigma_{\tau R}^{2}=\sigma_{\tau C(+)}^{2}=\sigma_{\tau C(-)}^{2}=\sigma_{\tau RC(+)}^{2}=\sigma_{\tau RC(-)}^{2}=0$$ (23)

and

$$\sigma_{C(-)}^{2}+\sigma_{RC(-)}^{2}+\sigma_{E(-)}^{2}=1.$$ (24)

This result can also be applied to original RM null or constrained unequal-variance RM null models, since they are specific applications of the unconstrained unequal-variance RM null model. In particular, it follows from Eqs. (5), (23), and (24) that a constrained unequal-variance null RM or an original RM null model is an RM identical-test model if it can be expressed by mixed linear model Eq. (1) with

$$\sigma_{\tau R}^{2}=\sigma_{\tau C(-)}^{2}=\sigma_{\tau RC(-)}^{2}=0$$ (25)

and constraint Eq. (24).

## 4 Examples of RM Null and RM Identical-Test Models

Table 1 illustrates the derivation of several RM identical-test models from RM null models using Eqs. (16)–(22). In row 1 of the table are the parameter values for one of the RM null models proposed by Roe and Metz.^5^ In row 2 are the variance components for the corresponding RM identical-test model, computed using Eqs. (16)–(22).

**Table 1:** Examples of RM null models and corresponding RM identical-test models.

Row	RM model	Model type	$\mu_{+}$	$\left(A_{z}\right)$ ^a^	$\sigma_{C(-)}^{2}$	$\sigma_{\tau C(-)}^{2}$	$\sigma_{RC(-)}^{2}$	$\sigma_{\varepsilon (-)}^{2}$	$\sigma_{C(+)}^{2}$	$\sigma_{\tau C(+)}^{2}$	$\sigma_{RC(+)}^{2}$	$\sigma_{\varepsilon (+)}^{2}$	$\sigma_{R}^{2}$	$\sigma_{\tau R}^{2}$
1	(a) Original^5^	Null	1.50	(0.856)	0.3	0.3	0.2	0.2	0.3	0.3	0.2	0.2	0.0055	0.0055
2		Identical-test	1.50	(0.856)	0.6	0.0	0.2	0.2	0.6	0.0	0.2	0.2	0.0110	0.0000
3	(b) Const. unequal var.^6^	Null	1.831	(0.856)	0.3	0.3	0.2	0.2	0.593	0.593	0.40	0.40	0.0082	0.0082
4		Identical-test	1.831	(0.856)	0.6	0.0	0.2	0.2	1.186	0.0	0.40	0.40	0.0164	0.0000
5	(c) Unconst. unequal var.^9^	Null	1.50	(0.826)	0.3	0.3	0.2	0.2	0.4	0.45	0.25	0.35	0.007	0.004
6		Identical-test	1.50	(0.826)	0.6	0.0	0.2	0.2	0.85	0.00	0.25	0.35	0.011	0.000

Notes: “Const.” = constrained; “Unconst.” = unconstrained; and “var.” = variance; *b* = 0.771 for the constrained unequal variance RM model, RM model (b).

a $A_{z}$ is equal to the median AUC across the reader population; the purpose of the parentheses is to indicate that it is not an RM model parameter used for simulating data, but rather is included to provide additional information about the model. It is computed using $A_{z}=\Phi (\mu_{+}/\sqrt{\sigma_{-}^{2}+\sigma_{+}^{2}})$, where $\sigma_{-}^{2}=\sigma_{C(-)}^{2}+\sigma_{\tau C(-)}^{2}+\sigma_{RC(-)}^{2}+\sigma_{\varepsilon (-)}^{2}=1$ and $\sigma_{+}^{2}=\sigma_{C(+)}^{2}+\sigma_{\tau C(+)}^{2}+\sigma_{RC(+)}^{2}+\sigma_{\varepsilon (+)}^{2}$.

Similarly, in row 3 are the parameter values for a constrained unequal variance null RM model given by Hillis,^6^ which has the same median AUC and the same variance components for random effects involving nondiseased cases as the original RM null model in row 1, but sets b=0.711 so that the median mean-to-sigma ratio^10^ will be 4.50. In row 4 are the corresponding identical-test parameter values, derived using Eqs. (16)–(22).

Finally, in row 5 is an unconstrained unequal variance null RM model^6^ with the corresponding identical-test model variance components, again derived using Eqs. (16)–(22), given in row 6.

## 5 Review and Comparison of Conventional OR, Unconstrained OR, and Gallas MRMC Methods

### 5.1 OR Method

The OR method assumes a test × reader factorial ANOVA model for AUC estimates and other reader performance measure estimates resulting from an MRMC study, with each AUC estimate corresponding to one reader using one of several tests (typically an imaging modality). Here we are assuming the study design discussed in the first paragraph of Sec. 1. Unlike a conventional ANOVA model, the errors are assumed to be correlated to account for correlation due to each reader evaluating the same cases.

The OR model is given as

$${\hat{\theta }}_{ij}=\mu_{\mathrm{OR}}+\tau_{i:\mathrm{OR}}+R_{j:\mathrm{OR}}+(\tau R{)}_{ij:\mathrm{OR}}+\varepsilon_{ij:\mathrm{OR}},$$ (26)

where $\mu_{\mathrm{OR}}$ is the fixed intercept term, $\tau_{i:\mathrm{OR}}$ denotes the fixed effect of test i, $R_{j:\mathrm{OR}}$ denotes the random effect of reader j, $(\tau R{)}_{ij:\mathrm{OR}}$ denotes the random test × reader interaction, and $\varepsilon_{ij:\mathrm{OR}}$ is the error term. The $R_{j:\mathrm{OR}}$ and $(\tau R{)}_{ij:\mathrm{OR}}$ are assumed to be mutually independent and normally distributed with zero means and respective variances $\sigma_{R:\mathrm{OR}}^{2}$ and $\sigma_{\tau R:\mathrm{OR}}^{2}$. (We include “OR” in effect and variance component subscripts to distinguish OR effects and variance components from similarly notated RM-model quantities.) The $\varepsilon_{ij:\mathrm{OR}}$ are assumed to be normally distributed with mean zero and variance $\sigma_{\varepsilon :\mathrm{OR}}^{2}$ and are assumed uncorrelated with the $R_{j:\mathrm{OR}}$ and $(\tau R{)}_{ij:\mathrm{OR}}$. Three possible error covariances are assumed:

$$\mathrm{Cov}(\varepsilon_{ij:\mathrm{OR}},\varepsilon_{i^{\prime }j^{\prime }:\mathrm{OR}})=\{\begin{array}{ll} {\mathrm{Cov}}_{1} & i\neq i^{\prime },j=j^{\prime }(\text{different test},\text{same reader})\\ {\mathrm{Cov}}_{2} & i=i^{\prime },j\neq j^{\prime }(\text{same test},\text{different reader})\\ {\mathrm{Cov}}_{3} & i\neq i^{\prime },j\neq j^{\prime }(\text{different test},\text{different reader}). \end{array}$$

The OR model assumes^11^

$${\mathrm{Cov}}_{1}\geq {\mathrm{Cov}}_{3},\;{\mathrm{Cov}}_{2}\geq {\mathrm{Cov}}_{3},\;{\mathrm{Cov}}_{3}\geq 0.$$ (27)

The OR model can alternatively be described with population correlations

$$r_{i}={\mathrm{Cov}}_{i}/\sigma_{\varepsilon }^{2},\;i=1,2,3,$$ (28)

instead of the covariances, i.e., with ${\mathrm{Cov}}_{i}$ replaced by $r_{i}\sigma_{\varepsilon }^{2}$, i=1,2,3.

These error variance-covariance parameters are typically estimated by averaging corresponding fixed-reader estimates computed using the jackknife,^12^^–^^14^ bootstrap,^14^^,^^15^ or the method proposed by DeLong et al.^16^ (DeLong), with DeLong only for empirical AUC estimates. These three estimation methods are consistent but are not unbiased. An unbiased error covariance method, which we will refer to as the “unbiased” method, was recently proposed by Hillis^17^ for use when empirical AUC is the outcome. This method utilizes the unbiased method fixed-reader method discussed by Gallas (Ref. 3, p. 362) for estimating the error variance [which Gallas notes is equivalent to the expressions given by Bamber (Ref. 18, p. 402)] and extensions of it for estimating the error covariances. OR analysis using this method is included in the freely available R software package MRMCaov.^19^

### 5.2 Conventional OR Test Statistic and Variance Estimate

The conventional OR test statistic for testing the null hypothesis of no test effect ($H_{0}:\tau_{1}=\ldots =\tau_{N_{T}}$) is given as

FOR=MS(T)MS(T*R)+max[NR(Cov^2−Cov^3),0], (29)

where MS(T*R)=1(NT−1)(NR−1)∑i=1NT∑j=1NR(θ^ij−θ^i•−θ^•j+θ^••)2, MS(T)=NRNT−1∑i=1NT(θ^i•−θ^••)2, $N_{T}\geq 2$ is the number of tests, $N_{R}$ is the number of readers and ${\hat{\mathrm{Cov}}}_{2}$ and ${\hat{\mathrm{Cov}}}_{3}$ are the ${\mathrm{Cov}}_{2}$ and ${\mathrm{Cov}}_{3}$ estimates. Here a subscript replaced by a dot indicates the average across the corresponding levels; e.g., θ^i•=∑j=1NRθ^ij/NR. Under $H_{0}$, F has an approximate F distribution with numerator degrees of freedom $N_{T}-1$ and denominator degrees of freedom^2^

ddfH≡{MS(T*R)+max[NR(Cov^2−Cov^3),0]}2[MS(T*R)]2/[(NT−1)(NR−1)]. (30)

For $N_{T}=2$ tests, Eq. (29) can be written in the form

$$F_{\mathrm{OR}}=\frac{({\hat{\theta }}_{1•}-{\hat{\theta }}_{2•}{)}^{2}}{{\hat{\mathrm{var}}}_{\mathrm{OR}}({\hat{\theta }}_{1•}-{\hat{\theta }}_{2•})},$$ (31)

where

$${\hat{\mathrm{var}}}_{\mathrm{OR}}({\hat{\theta }}_{1•}-{\hat{\theta }}_{2•})=\frac{2}{N_{R}}\{\mathrm{MS}(T*R)+\max[N_{R}({\hat{\mathrm{Cov}}}_{2}-{\hat{\mathrm{Cov}}}_{3}),0]\},$$ (32)

is the OR estimate for the variance of ${\hat{\theta }}_{1•}-{\hat{\theta }}_{2•}$.

Note that Eqs. (29)–(32) incorporate the error-covariance constraints given in Eq. (27). We will sometimes refer to these as the “conventional OR” F statistics, denominator degrees of freedom estimate and variance estimate, to distinguish them from the unconstrained versions of these statistics discussed below.

### 5.3 Unconstrained OR Test Statistics and Variance Estimate

The importance of the OR constraints given in Eq. (27) will be demonstrated by simulations in Sec. 6. In this section the OR test statistics and variance estimate are defined without constraints Eq. (27) imposed. Use of these unconstrained test statistics in place of Eqs. (29)–(32) will be called the “unconstrained OR” method.

The unconstrained OR test statistics, denominator degrees of freedom and variance are given as

FOR;unconstrained=MS(T)MS(T*R)+NR(Cov^2−Cov^3), (33)

ddfH;unconstrained={MS(T*R)+NR(Cov^2−Cov^3),0}2[MS(T*R)]2/[(NT−1)(NR−1)], (34)

and

$$F_{\mathrm{OR};\mathrm{unconstrained}}=\frac{({\hat{\theta }}_{1•}-{\hat{\theta }}_{2•}{)}^{2}}{{\hat{\mathrm{var}}}_{\mathrm{OR};\mathrm{unconstrained}}({\hat{\theta }}_{1•}-{\hat{\theta }}_{2•})},$$ (35)

where

var^OR;unconstrained(θ^1•−θ^2•)=2NR{MS(T*R)+NR(Cov^2−Cov^3)}. (36)

Note that

FOR=FOR;unconstrained  if  Cov^2−Cov^3≥0, (37)

and that Eq. (35) is not defined if Eq. (36) is not positive.

### 5.4 Equivalence of Gallas and Unconstrained OR F Statistics

When the outcome is the empirical AUC and there are two tests, Hillis^17^ has shown that the Gallas method F statistic for testing the null hypothesis of no difference in test AUCs is equivalent to the unconstrained OR method F statistic Eq. (35) when the unbiased covariance estimation method is used to compute ${\hat{\mathrm{Cov}}}_{2}$ and ${\hat{\mathrm{Cov}}}_{3}$. However, the Gallas denominator degrees of freedom estimate differs from the conventional and unconstrained OR denominator degrees of freedom estimates.

### 5.5 Relationship of OR Model and RM Identical-Test Model

Hillis^9^ derived the OR parameters for the distribution of empirical AUC estimates simulated using the unconstrained unequal-variance RM model. I show in Appendix A that it follows from these results that for data simulated from the unconstrained unequal-variance RM identical-test model

$$\mu_{\mathrm{OR}}+\tau_{1:\mathrm{OR}}=\mu_{\mathrm{OR}}+\tau_{2:\mathrm{OR}},$$ (38)

$${\mathrm{Cov}}_{2}={\mathrm{Cov}}_{3},$$ (39)

$$\sigma_{\tau R:\mathrm{OR}}^{2}=0.$$ (40)

These results are intuitive. The first result states that the expected AUCs (as given by $\mu_{\mathrm{OR}}+\tau_{i:\mathrm{OR}}$ for test i) must be the same for each test and the second result states that ${\mathrm{Cov}}_{2}$ and ${\mathrm{Cov}}_{3}$ must be equal, which makes sense since for equal tests the covariances have the same definition. To understand the third result, we note that it can be shown [Ref. 9, p. 2069] that $\sigma_{\tau R}^{2}$ is equal to half of the variance of the within-reader differences of the expected AUCs; under the assumption of the identical-test RM model, these differences are zero, and hence $\sigma_{\tau R}^{2}=0$.

## 6 Simulation Studies Comparing Conventional and Unconstrained OR Based on RM Null and Identical-Test Models

### 6.1 Simulation Study Using Tables 1(a) and 1(b) RM Null and Identical-Test Models

Multi-reader rating data for five readers, each reading the same cases under two tests, were simulated based on the original RM null and corresponding RM identical-test models, and on the constrained unequal variance RM null and corresponding RM identical-test models, given in Tables 1(a) and 1(b), respectively. (Results based on the Table 1(c) model are omitted from Table 1 for brevity and because the Table 1(c) RM null model parameter values, unlike the other two RM null model parameter values, have not been previously suggested in the literature.) For each model, 5000 simulated MRMC samples were generated for case sample sizes of 25/25 and 50/50 each, where “25/25” indicates 25 nondiseased and 25 diseased cases. The empirical AUC was computed for each simulated MRMC sample with OR error covariances estimated using the unbiased error-covariance method. The null hypothesis of equal test AUCs, versus the two-sided alternative hypothesis, was tested at the 0.05 significance level using both the conventional and unconstrained OR test statistics, given by Eqs. (31) and (35). Results of the simulations, presented in Table 2, include the empirical type I error rate; the proportion of samples having negative variance estimates, as defined by Eq. (32) or Eq. (36); and the proportion of negative values for ${\hat{\mathrm{Cov}}}_{2}-{\hat{\mathrm{Cov}}}_{3}$.

**Table 2:** OR analysis results using the unbiased covariance method, for 5 readers reading the same cases under both tests, with 5000 MRMC samples simulated from Table 1 RM null models (a) and (b) and their corresponding identical-test models for each case size combination.

No. of cases	RM model	Model type	OR F statistic	Rates		
				$\hat{\mathrm{var}}({\hat{\theta }}_{1}-{\hat{\theta }}_{2})<0$	Type I error	${\hat{\mathrm{Cov}}}_{2}-{\hat{\mathrm{Cov}}}_{3}<0$
25/25	(a) Original	Null	Conventional	0	0.050	0
			Unconstrained	0	0.050	0
		Identical-test	Conventional	0	0.043	0.52
			Unconstrained	0.039	N/A	0.52
	(b) Const. unequal var.	Null	Conventional	0	0.048	0
			Unconstrained	0	0.048	0
		Identical-test	Conventional	0	0.033	0.53
			Unconstrained	0.049	N/A	0.53
50/50	(a) Original	Null	Conventional	0	0.053	0
			Unconstrained	0	0.053	0
		Identical-test	Conventional	0	0.046	0.52
			Unconstrained	0.026	N/A	0.52
	(b) Const. unequal var.	Null	Conventional	0	0.051	0
			Unconstrained	0	0.051	0
		Identical-test	Conventional	0	0.046	0.53
			Unconstrained	0.031	N/A	0.53

Notes: see Table 1 for definitions of “RM model” and “Model type”; OR F statistic = “Conventional” if the OR constrained F statistic Eq. (32) is used and = “Unconstrained” if the unconstrained F statistic Eq. (33) is used; “25/25" indicates 25 nondiseased and 25 diseased cases; “type I error” is the proportion of samples where the null hypothesis of no test effect is rejected; “N/A” stands for “not applicable” and indicates that the empirical type I error rate could not be computed because the variance of the test statistic, computed using Eq. (36), was negative for some samples. Note that although ${\hat{\mathrm{Cov}}}_{2}-{\hat{\mathrm{Cov}}}_{3}$ is not constrained in this table, in the computation of the conventional OR variance it is constrained to be nonnegative.

If the variance estimate was negative, the type I error rate could not be computed because the test statistic Eq. (31) or Eq. (35), which is required for deciding whether to accept or reject the null hypothesis of equal test AUCs, was not defined for all the simulated samples; this situation is indicated in Table 2 by “NA” (not applicable).

#### 6.1.1 RM null model results

We see from Table 2 that when model type = “null,” the empirical type I error rates are the same for the conventional and constrained OR methods, with the type I rates varying between 0.048 and 0.051. That these rates are the same can be explained by Eq. (37) and by the nonnegativity of ${\hat{\mathrm{Cov}}}_{2}-{\hat{\mathrm{Cov}}}_{3}$ for all the samples (as indicated in the last column).

#### 6.1.2 Identical-test model results

In contrast to the null model results reported above, we see in Table 2 that the identical-test model type I error rates depend on whether the conventional or unconstrained OR method was used.

##### Conventional OR results

For the identical-test models the conventional OR type I error rates vary between 0.033 and 0.046 with no negative variance estimates.

##### Unconstrained OR results

For the identical-test models, all of the unconstrained OR type I error rates were undefined (as indicated by “NA” in Table 2) because of negative variance estimates. For the original RM identical-test and constrained unequal variance RM identical-test models, respective negative unconstrained OR variance rates were 0.039 and 0.049 for 25/25 samples and 0.026 and 0.031 for 50/50 samples. Note that these negative variance estimate rates apply also to the Gallas F statistic, since it is the same as the unconstrained OR F statistic, as discussed in Sec. 5.4.

### 6.2 Simulation Study Using Original Roe and Metz Null Models and Corresponding Identical-Test Null Models

In the original Roe and Metz^5^ paper, four different variance component “structures,” denoted as “HL,” “LL,” “HH,” and “LH,” are given for $\mu_{+}=0.75$, 1.5, and 2.50, resulting in twelve different parameter combinations. In the upper half of Table 3 are the four variance component structures for $\mu_{+}=1.5$. In the lower half of Table 3 are the corresponding RM identical-test model structures that result from application of Eqs. (16)–(22) to the structures in the upper half of Table 3. (See Table 8 in Appendix B for a similar table that includes all twelve parameter combinations and corresponding RM identical-test parameter specifications.)

**Table 3:** Subset of original 12 sets of Roe and Metz^5^ (RM) null simulation model parameter values and corresponding RM identical-test model parameter values. The Table 4 simulation results are based on these parameter values. The complete set of 12 sets of parameter values is included in Table 8.

Structure	$\mu_{+}$	$A_{z}$	$\sigma_{C}^{2}$	$\sigma_{\tau C}^{2}$	$\sigma_{RC}^{2}$	$\sigma_{\varepsilon }^{2}$	$\sigma_{R}^{2}$	$\sigma_{\tau R}^{2}$
(a) Original RM model parameter values								
HL	1.5	0.856	0.3	0.3	0.2	0.2	0.0055	0.0055
LL	1.5	0.856	0.1	0.1	0.2	0.6	0.0055	0.0055
HH	1.5	0.856	0.3	0.3	0.2	0.2	0.0300	0.0300
LH	1.5	0.856	0.1	0.1	0.2	0.6	0.0300	0.0300
(b) Corresponding RM identical-test model parameter values								
HL	1.5	0.856	0.6	0.0	0.2	0.2	0.0110	0.0000
LL	1.5	0.856	0.2	0.0	0.2	0.6	0.0110	0.0000
HH	1.5	0.856	0.6	0.0	0.2	0.2	0.0600	0.0000
LH	1.5	0.856	0.2	0.0	0.2	0.6	0.0600	0.0000

Notes: $\mu_{+}$ is the median and mean separation of the normal and abnormal DV distributions across the reader population, and $A_{z}=\Phi (\mu_{+}/\sqrt{2})$ is the median reader-specific true area under the ROC curve.

For each parameter combination in Table 3, 2000 MRMC samples were simulated for each of 6 combinations of 3 reader levels (3, 5, and 10 readers) and 2 sample size levels (25/25 and 50/50). Each set of 2000 samples was analyzed using the conventional and unconstrained OR methods, using both unbiased and DeLong error-covariance estimates.

For each error covariance method and model type (null or identical-test), Table 4 presents the analysis results for each reader and sample size combination, averaged across the four structures in Table 3. For example, the type I error of 0.061 in the first row of Table 4 is the average of four empirical type I error rates, corresponding to the four original RM null model structures, resulting from performing a conventional OR analysis using the DeLong covariance method on each of 2000 simulated MRMC samples for each structure, with each simulated MRMC sample containing rating data from 3 readers reading 25 nondiseased and 25 diseased cases. (For brevity, averages of the four empirical type I error rates are reported rather than the rates for each separate structure, since the averages are sufficient to reveal the problem of negative variances with the unconstrained OR method.)

**Table 4:** Conventional and unconstrained OR analysis results using the DeLong and unbiased error covariance methods, for MRMC samples simulated from the original RM null model and the corresponding RM identical-test model parameter values given in Table 3. Readers read the same cases under both tests. For each combination of structure (HL, LL, HH, or LH), error covariance method (Delong or unbiased), readers (3, 5, or 10) and case sample sizes (25/25 or 50/50), 2000 MRMC samples were simulated and analyzed using both conventional and unconstrained OR with empirical AUC being the outcome. This table presents those analysis results averaged across the four structures. For example, the conventional OR type I error of 0.061 in the first line is the average of the four conventional OR empirical type I error statistics computed for each of the four parameter structures in Table 3, based on 2000 simulated MRMC samples for each structure for 3 readers each reading 25 nondiseased and 25 diseased cases.

Row	Covariance	RM model	Cases	Readers	N	Conventional OR		Unconstrained OR		Conventional and unconstrained OR				
						var < 0	Type I	var < 0	Type I	${\mathrm{Cov}}_{2}$	${\mathrm{Cov}}_{3}$	$\sigma_{\tau R;\mathrm{OR}}^{2}$	${\mathrm{AUC}}_{1}$	${\mathrm{AUC}}_{2}$
1	DeLong	Null	25/25	3	4	0.0000%	0.061	0.8625%	NA	0.0009	0.0004	0.0008	0.852	0.852
2				5	4	0.0000%	0.049	0.0250%	NA	0.0010	0.0004	0.0009	0.851	0.851
3				10	4	0.0000%	0.051	0.0000%	0.051	0.0010	0.0004	0.0009	0.851	0.851
4			50/50	3	4	0.0000%	0.064	0.1875%	NA	0.0005	0.0002	0.0010	0.851	0.852
5				5	4	0.0000%	0.049	0.0250%	NA	0.0005	0.0002	0.0009	0.851	0.852
6				10	4	0.0000%	0.045	0.0000%	0.045	0.0005	0.0002	0.0010	0.851	0.851
7		Identical-test	25/25	3	4	0.0000%	0.056	10.3630%	NA	0.0009	0.0009	-0.0001	0.852	0.852
8				5	4	0.0000%	0.043	4.6500%	NA	0.0010	0.0010	-0.0001	0.851	0.851
9				10	4	0.0000%	0.041	1.0630%	NA	0.0010	0.0010	-0.0001	0.851	0.851
10			50/50	3	4	0.0000%	0.053	7.1630%	NA	0.0005	0.0005	0.0000	0.852	0.852
11				5	4	0.0000%	0.043	2.3000%	NA	0.0005	0.0005	0.0000	0.851	0.851
12				10	4	0.0000%	0.042	0.3000%	NA	0.0005	0.0005	0.0000	0.852	0.852
13	Unbiased	Null	25/25	3	4	0.0000%	0.061	0.8375%	NA	0.0009	0.0004	0.0009	0.852	0.852
14				5	4	0.0000%	0.049	0.0250%	NA	0.0009	0.0004	0.0010	0.851	0.851
15				10	4	0.0000%	0.052	0.0000%	0.052	0.0009	0.0004	0.0010	0.851	0.851
16			50/50	3	4	0.0000%	0.064	0.1875%	NA	0.0005	0.0002	0.0010	0.851	0.852
17				5	4	0.0000%	0.049	0.0250%	NA	0.0005	0.0002	0.0009	0.851	0.852
18				10	4	0.0000%	0.046	0.0000%	0.046	0.0005	0.0002	0.0010	0.851	0.851
19		Identical-test	25/25	3	4	0.0000%	0.056	10.3380%	NA	0.0009	0.0009	0.0000	0.852	0.852
20				5	4	0.0000%	0.044	4.5750%	NA	0.0010	0.0010	0.0000	0.851	0.851
21				10	4	0.0000%	0.041	1.0380%	NA	0.0010	0.0010	0.0000	0.851	0.851
22			50/50	3	4	0.0000%	0.053	7.1630%	NA	0.0005	0.0005	0.0000	0.852	0.852
23				5	4	0.0000%	0.043	2.3000%	NA	0.0005	0.0005	0.0000	0.851	0.851
24				10	4	0.0000%	0.042	0.2880%	NA	0.0005	0.0005	0.0000	0.852	0.852

Notes: “covariance” = error covariance method used with the OR method; “N” = number of parameter strutures in Table 3 that results are averaged across; “25/25" indicates 25 nondiseased and 25 diseased cases; “type I ” is the empirical type I error rate; “var < 0" is the proportion of samples where the variance estimate for the difference of the reader-averaged test AUCs is negative, and hence the OR F statistic is not defined; ${\mathrm{Cov}}_{2}$, ${\mathrm{Cov}}_{3}$, and $\sigma_{\tau R:\mathrm{OR}}^{2}$ are OR parameter estimates (which are the same for the conventional and unconstrained OR methods); ${\mathrm{AUC}}_{1}$ and ${\mathrm{AUC}}_{2}$ are the empirical AUCs for tests 1 and 2, respectively.

Results of the simulations, presented in Table 4, include the empirical type I error rate and the negative-variance rate. The negative-variance rate is the proportion of samples having negative variance estimates, as defined by Eq. (32) or Eq. (36), for both the conventional and unconstrained OR methods. As in Table 2, a value of “NA” for the type I rate indicates at least one sample had a negative variance estimate, and hence an undefined type I error rate. Table 4 also includes the averages of the empirical AUC estimates for tests 1 and 2 and the averages of the OR estimates for ${\mathrm{Cov}}_{2}$, ${\mathrm{Cov}}_{3}$, and $\sigma_{TR;\mathrm{OR}}^{2}$; these last three estimates depend on the OR error covariance method but not on the use of conventional or unconstrained OR.

From Table 4, I make the following remarks.

1. **OR results are similar for DeLong and unbiased covariance methods.** For the original RM null model, a comparison of rows 1 to 6 with 13 to 18 shows only slight differences between the DeLong and corresponding unbiased covariance method results. Similarly, there are only slight differences between the DeLong and unbiased covariance method results for the RM identical-test model, as can be seen from comparing rows 7 to 12 with 19 to 24.
2. **Conventional OR has acceptable type I rates and no negative variances.** For the conventional OR method, type I error averages (across the four structures) are between 0.041 to 0.064, with the overall average type I rate average (not shown) equal to 0.050 for both the unbiased and DeLong methods. As shown in the “var < 0” column, none of the conventional OR variance estimates, computed using Eq. (32), were negative as expected, since it is impossible for Eq. (32) to be negative.
3. **Unconstrained OR type I errors are undefined for most parameter combinations because of negative variances.** For the unconstrained OR method using the DeLong covariance estimation method, 10 of the 12 sets of 8000 samples (2000 samples × 4 structures) resulted in negative variance estimates (see rows 1-12, “Unconstrained OR” columns). As a result, type I error was not defined for any of the 6 identical-test parameter combinations, and only for 2 of the 6 null model combinations (for 10 readers and 50/50 cases, rows 3 and 6). For the identical-test model, the negative-variance (“var < 0”) rates for the unconstrained OR method range from 0.3% to 10.4%, with rates being higher for smaller numbers of cases and readers. For the null model the rates were much smaller, with the highest negative-variance rate equal to 0.9%; again, rates were higher for smaller numbers of cases and readers. The above comments also apply to the results for the OR method using the unbiased covariance estimation method in rows 13 to 24.
4. **OR parameter relationships for an identical-test model are validated.** For the identical-test models, the ${\mathrm{Cov}}_{2}$ and ${\mathrm{Cov}}_{3}$ estimates are approximately equal and the OR test-by-reader variance component ($\sigma_{TR:\mathrm{OR}}^{2})$ estimates are approximately zero, regardless of which covariance method is used. Also, the AUC estimates are approximately equal for each test. These empirical results validate the OR parameter relationships given by Eqs. (38)–(40) in Sec. 5.5 for identical-test models.

## 7 Understanding How a Negative Variance Occurs

We can rewrite Eq. (36) in the form

var^OR;unconstrained(θ^1•−θ^2•)=A+B (41)

where

A=2NRMS(T*R),B=2(Cov^2−Cov^3). (42)

The A term will never be negative because MS(T*R) cannot be negative. Thus var^OR;unconstrained(θ^1•−θ^2•) can be negative only if ${\hat{\mathrm{Cov}}}_{2}-{\hat{\mathrm{Cov}}}_{3}$ is sufficiently negative to result in B<−A. For the unconstrained unequal-variance RM identical-test model, ${\hat{\mathrm{Cov}}}_{2}$ and ${\hat{\mathrm{Cov}}}_{3}$ have the same distributions; thus $E({\hat{\mathrm{Cov}}}_{2}-{\hat{\mathrm{Cov}}}_{3})=0$ and ${\hat{\mathrm{Cov}}}_{2}-{\hat{\mathrm{Cov}}}_{3}$ has a symmetric distribution about 0. It follows that B will be negative with probability 0.5, which is in agreement with the results in Table 2 where the negative ${\hat{\mathrm{Cov}}}_{2}-{\hat{\mathrm{Cov}}}_{3}$ rates are ∼50%.

It has been shown by Hillis,^17^ under the assumption of the unconstrained unequal-variance RM model, that

$$E(A)=\frac{2}{N_{R}}[(\sigma_{\varepsilon :\mathrm{OR}}^{2}-{\mathrm{Cov}}_{1})+\sigma_{\tau R:\mathrm{OR}}^{2}-({\mathrm{Cov}}_{2}-{\mathrm{Cov}}_{3})]\;=\frac{2\sigma_{\varepsilon :\mathrm{OR}}^{2}}{N_{R}}[(1-r_{1})+\sigma_{\tau R:\mathrm{OR}}^{2}/\sigma_{\varepsilon :\mathrm{OR}}^{2}-(r_{2}-r_{3})].$$ (43)

Here and elsewhere in this section I often express ${\mathrm{Cov}}_{i}=r_{i}\sigma_{\varepsilon :\mathrm{OR}}^{2}$, i=1,2,3 because it has been shown^9^ that these correlations remain approximately constant for a given RM model across different reader sample sizes and case sample sizes, making them easy to interpret.

To simplify the discussion, I now assume that the estimates ${\hat{\mathrm{Cov}}}_{2}-{\hat{\mathrm{Cov}}}_{3}$ are unbiased, which is the case when the unbiased error-covariance method is used with OR. Making this assumption, it follows from Eqs. (41)–(43) that

E[var^OR;unconstrained(θ^1•−θ^2•)]=2σε:OR2NR[(1−r1)+στR:OR2/σε:OR2+(NR−1)(r2−r3)]. (44)

Although Eq. (44) assumes unbiased ${\mathrm{Cov}}_{2}$ and ${\mathrm{Cov}}_{3}$ estimates, typically we expect the right side of Eq. (44) to approximate the left side when a reasonable alternative error covariance estimation method is used, such as the jackknife or DeLong method.

From Eq. (44) it follows that E[var^OR;unconstrained(θ^1•−θ^2•)] increases as $\left(r_{2}-r_{3}\right)$ and $\sigma_{\tau R:\mathrm{OR}}^{2}$ increase, assuming all other parameters in the model remain the same. Thus, recalling that for an RM identical-test model $(r_{2}-r_{3})=0$ and $\sigma_{\tau R:\mathrm{OR}}^{2}=0$, it seems likely that the probability of a negative variance will decrease as $\left(r_{2}-r_{3}\right)$ or $\sigma_{\tau R:\mathrm{OR}}^{2}$ increase. On the other hand, because Eq. (44) does not depend on the difference of the AUCs, as shown by the omission of $\tau_{1:\mathrm{OR}}$ and $\tau_{2:\mathrm{OR}}$, there is no indication that the probability of a negative variance will decrease or increase as the magnitude of the AUC difference increases.

## 8 Simulation Studies for Examining Effects of ${\mathrm{AUC}}_{1}-{\mathrm{AUC}}_{2},r_{2}-r_{1}$ and $\sigma_{\tau R;\mathrm{OR}}^{2}$ on Negative Variance Rates

### 8.1 Purpose

The simulations in Sec. 6 established the usefulness of the identical-test RM model for detecting the negative variance problem inherent in using the unconstrained OR procedure. A natural follow-up question to ask is, “to what extent does the unconstrained OR procedure have this problem when the conditions of the identical-test RM model are not exactly satisfied?” The purpose of this section is to empirically address this question by simulating data from RM simulation models that are not identical-test RM models.

As discussed in Sec. 5.5, data simulated from an identical-test RM model results in AUC estimates such that three conditions are true: (1) the tests have equal expected AUCs; (2) the OR ${\mathrm{Cov}}_{2}$ and ${\mathrm{Cov}}_{3}$ parameters are equal, or equivalently, $r_{2}-r_{3}=0$ where $r_{2}$ and $r_{3}$ are the OR correlations defined by Eq. (28); and (3) the OR test-by-reader interaction variance component $\sigma_{\tau R}^{2}$ is zero. These conditions are implied by Eqs. (38)–(40). In this section I simulate data from RM models that have been formulated such that not all of these conditions are true, and thus none of the simulation models are identical-test RM models. The results of these simulations will allow us to answer the question posed in the previous paragraph, as well as to provide support for the conjectures in Sec. 7 regarding the associations between each of the three conditions and the negative variance rate.

### 8.2 Simulations

#### 8.2.1 Overview

Data are simulated that result in OR distributions with parameter values similar to those estimated for two real datasets that are analyzed by the unconstrained OR method with empirical AUC as the outcome and using the unbiased error-covariance method. In each of the 2 examples, 10,000 MRMC samples are simulated from 8 different constrained unequal-variance RM models, with each corresponding empirical AUC distribution corresponding to one of eight possible combinations of 2 different levels for $r_{2}-r_{3},\sigma_{\tau R:\mathrm{OR}}^{2}$ and ${\mathrm{AUC}}_{1}-{\mathrm{AUC}}_{2}$. The two levels are 0.01 and 0.04 for $\left(r_{2}-r_{3}\right)$, 0.0000 and 0.0002 for $\sigma_{\tau R}^{2}$, and 0.00 and 0.04 for ${\mathrm{AUC}}_{1}-{\mathrm{AUC}}_{2}$ (note that ${\mathrm{AUC}}_{i}=\mu_{\mathrm{OR}}+\tau_{i:\mathrm{OR}}$). All of these values are representative of real datasets. The case and reader sample sizes for the simulated MRMC samples are the same as for the original datasets. Although ${\hat{r}}_{2}-{\hat{r}}_{3}<0$ for both of the original datasets, a negative value for $r_{2}-r_{3}$ is not included as one of the study design parameters because the OR model assumes $r_{2}-r_{3}\geq 0$.

#### 8.2.2 Example: simulations based on Kundel dataset

Kundel et al.^20^ compared reader AUCs for hard-copy and soft-copy computed radiograph chest images selected randomly from a medical intensive care unit. Four radiologists blindly read both types of images obtained from the same patients. Six months separated the end of the hard-copy readings and the start of the soft-copy readings. A five-point ordinal scale was used to rate the likelihood of the presence of the condition (which we will consider to be the disease) implied by the reason for requesting the corresponding examination. Ninety-five images, consisting of 29 diseased and 66 nondiseased images, were read under each test condition. The difference of the empirical AUC estimates was 0.0375 (p=0.0916) and ${\hat{r}}_{2}-{\hat{r}}_{3}$ was 0.38−0.40=−0.02, computed from a conventional OR analysis using the unbiased covariance method. The unconstrained variance estimate was not negative.

The OR parameter estimates for the original data, using the unbiased covariance method, are shown in Table 5(a). In Table 5(b) are eight sets of parameter values similar to the original data estimates, corresponding to the eight possible combinations of the levels of $r_{2}-r_{3},\sigma_{\tau R}^{2}$ and ${\mathrm{AUC}}_{1}-{\mathrm{AUC}}_{2}$. Table 5(c) presents constrained unequal-variance RM model parameter values that result in simulated data that can be described by the OR parameters in Table 5(b); these were computed using the algorithm developed by Hillis et al.^21^ Because some of these RM models are not null models, $\mu_{\mathrm{pos}}$ in Eq. (1) is replaced by $\mu_{i},i=1,2$; thus $\mu_{i}$ is the expected difference in the means for the diseased and nondiseased DV distributions for test i.

**Table 5:** Simulations based on Kundel^20^ dataset showing effects of $r_{2}-r_{3}$, $\sigma_{\tau R:\mathrm{OR}}^{2}$ and ${\mathrm{AUC}}_{1}-{\mathrm{AUC}}_{2}$ on negative variance rates. In this study, 4 readers read the same 29 diseased and 66 nondiseased cases.

(a) OR estimates for original Kundel^20^ data set computed using the unbiased covariance method with empirical AUC as the outcome. The study included 4 readers and 29 diseased and 66 nondiseased cases.									
${\hat{\mathrm{AUC}}}_{1}$	${\hat{\mathrm{AUC}}}_{2}$	${\hat{\sigma }}_{R:\mathrm{OR}}^{2}$	${\hat{\sigma }}_{\tau R:\mathrm{OR}}^{2}$	${\hat{\sigma }}_{\varepsilon :\mathrm{OR}}^{2}$	${\hat{r}}_{1}$	${\hat{r}}_{2}$	r^3		
0.804	0.841	0.000734	0.00000	0.00215	0.508	0.384	0.404		
(b) OR parameter values describing simulated data sets.									
${\mathrm{AUC}}_{1}$	${\mathrm{AUC}}_{2}$	$\sigma_{R:\mathrm{OR}}^{2}$	$\sigma_{\tau R:\mathrm{OR}}^{2}$	$\sigma_{\varepsilon :\mathrm{OR}}^{2}$	$r_{1}$	$r_{2}$	$r_{3}$		
0.800	0.840	0.000734	0.00000	0.00215	0.510	0.390	0.380		
0.820	0.820	0.000734	0.00000	0.00215	0.510	0.390	0.380		
0.800	0.840	0.000734	0.00000	0.00215	0.510	0.405	0.365		
0.820	0.820	0.000734	0.00000	0.00215	0.510	0.405	0.365		
0.800	0.840	0.000734	0.00020	0.00215	0.510	0.390	0.380		
0.820	0.820	0.000734	0.00020	0.00215	0.510	0.390	0.380		
0.800	0.840	0.000734	0.00020	0.00215	0.510	0.405	0.365		
0.820	0.820	0.000734	0.00020	0.00215	0.510	0.405	0.365		
(c) Parameter values for constrained unequal-variance RM models used to simulate MRMC data sets corresponding to OR parameters in (b). Because some of these are not null models, $\mu_{\mathrm{pos}}$ in Eq. (1) has been replaced by $\mu_{i},i=1,2$.									
$\mu_{1}$	$\mu_{2}$	$\sigma_{R}^{2}$	$\sigma_{\tau R}^{2}$	$\sigma_{C}^{2}$	$\sigma_{TC}^{2}$	$\sigma_{RC}^{2}$	$\sigma_{\varepsilon }^{2}$	b	
1.209354	1.428970	0.011075	0	0.442032	0.006772	0.1303248	0.4208705	0.979343	
1.312015	1.312015	0.010902	0	0.438347	0.010547	0.1293156	0.4217907	0.984081	
1.209354	1.428970	0.011075	0	0.425999	0.038545	0.1463586	0.3890982	0.979343	
1.312015	1.312015	0.010902	0	0.422441	0.042189	0.1452217	0.3901491	0.984081	
1.212481	1.432665	0.011132	0.002879	0.442880	0.006785	0.1305745	0.4197602	0.977088	
1.315516	1.315516	0.010960	0.002970	0.439219	0.010568	0.1295731	0.4206395	0.981743	
1.212481	1.432665	0.011132	0.002879	0.426816	0.038619	0.1466390	0.3879270	0.977088	
1.315516	1.315516	0.010960	0.002970	0.423282	0.042273	0.1455109	0.3889350	0.981743	
(d) Estimates corresponding to OR parameters in (b) resulting from OR analysis of AUC data simulated using the RM models in (c)									
${\hat{\mathrm{AUC}}}_{1}$	${\hat{\mathrm{AUC}}}_{2}$	${\hat{\sigma }}_{R:\mathrm{OR}}^{2}$	${\hat{\sigma }}_{\tau R:\mathrm{OR}}^{2}$	${\hat{\sigma }}_{\varepsilon :\mathrm{OR}}^{2}$	${\hat{r}}_{1}$	${\hat{r}}_{2}$	${\hat{r}}_{3}$	var < 0 rate	$r_{2}<r_{3}$ rate
0.800	0.840	0.000722	0.00002	0.00215	0.510	0.390	0.380	4.2%	40.4%
0.820	0.820	0.000728	0.00001	0.00215	0.510	0.389	0.379	3.3%	40.2%
0.800	0.840	0.00074	0.00001	0.00214	0.511	0.405	0.364	1.0%	13.6%
0.820	0.820	0.000736	-0.00001	0.00216	0.509	0.405	0.365	1.0%	15.2%
0.799	0.839	0.000761	0.00018	0.00216	0.510	0.390	0.380	3.2%	40.5%
0.820	0.820	0.000743	0.00021	0.00215	0.510	0.391	0.381	3.1%	39.7%
0.800	0.840	0.000755	0.00019	0.00215	0.511	0.404	0.365	0.8%	14.4%
0.820	0.819	0.000745	0.00021	0.00216	0.510	0.406	0.365	0.8%	13.7%

Note: “(var < 0) rate” = proportion of samples where var^OR;unconstrained(θ^1•−θ^2•)<0.

Table 5(d) presents the estimates of the OR parameter estimates and the negative variance and negative ${\hat{r}}_{2}-{\hat{r}}_{3}$ rates computed from the simulated data, based on the RM models in Table 5(c). The excellent agreement between Tables 5(b) and 5(d) confirms that the RM model parameter values in Table 5(c) were appropriately chosen. In Table 5(d) negative variance rates range between 0.8% and 4.2% and negative ${\hat{r}}_{2}-{\hat{r}}_{3}$ rates range between 14% and 41%.

**Fig. 1:**
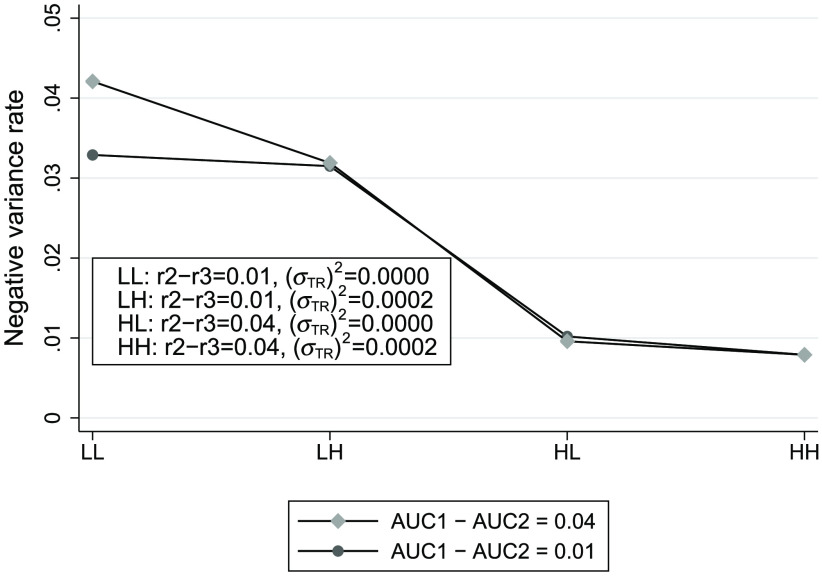
Negative unconstrained OR AUC-difference variance rates, as defined by Eq. (36), for different combinations of OR parameter values. RM model parameter values used for simulating the data are similar to estimates from an OR analysis of the Kundel et al.^20^ data. There were 10,000 simulated MRMC samples for each set of RM parameter values, with each sample corresponding to 4 readers reading the same 29 diseased and 66 nondiseased images.

Figure 1 displays a plot of the negative variance rate for each combination of the true values of $r_{2}-r_{3},\sigma_{\tau R}^{2}$ and ${\mathrm{AUC}}_{1}-{\mathrm{AUC}}_{2}$. The labels on the x-axis indicate the levels of $r_{2}-r_{3}$ and $\sigma_{\tau R}^{2}$, with “LL” indicating both at the lowest level, “LH” indicating the low level of $r_{2}-r_{3}$ and the high level of $\sigma_{\tau R}^{2}$, etc. From Fig. 1, we see that higher negative variance rates are associated with lower levels of both $r_{2}-r_{3}$ and $\sigma_{\tau R:\mathrm{OR}}^{2}$, in agreement with the conjectures in the previous section. The effect of ${\mathrm{AUC}}_{1}-{\mathrm{AUC}}_{2}$ is minimal except when both $\left(r_{2}-r_{3}\right)$ and $\sigma_{\tau R:\mathrm{OR}}^{2}$ are at their lowest levels, as shown by the first pair of points; for this situation the negative variance rate is higher for the larger magnitude of ${\mathrm{AUC}}_{1}-{\mathrm{AUC}}_{2}$. As noted in the previous section, there was no indication from Eq. (44) as to whether there would be any effect from ${\mathrm{AUC}}_{1}-{\mathrm{AUC}}_{2}$.

#### 8.2.3 Example: simulations based on Franken dataset

Franken et al.^22^ compared the diagnostic accuracy of interpreting clinical neonatal radiographs using a picture archiving and communication system workstation versus plain film. The case sample consisted of 100 chest or abdominal radiographs (67 abnormal and 33 normal). The readers were four radiologists with considerable experience in interpreting neonatal examinations. The readers indicated whether each patient had normal or abnormal findings and their degree of confidence in this judgment using a 5-point ordinal scale. The difference of the empirical AUC estimates was 0.0109 (p=0.1188) and ${\hat{r}}_{2}-{\hat{r}}_{3}$ was 0.32−.0.34=−0.02, computed from a conventional OR analysis using the unbiased covariance method. The unconstrained variance estimate was negative.

Table 6 gives results for this dataset in the same format as Table 5. Similar to Table 5, there is excellent agreement between Tables 6(b) and 6(d) that confirms that the RM model parameter values in Table 6(c) were appropriately chosen. In Table 6(d), negative variance rates range between 0.3% and 2.2% and negative $r_{2}-r_{3}$ rates range between 7% and 36%.

**Table 6:** Simulations based on Franken et al.^22^ dataset showing effects of $r_{2}-r_{3}$, $\sigma_{\tau R:\mathrm{OR}}^{2}$, and ${\mathrm{AUC}}_{1}-{\mathrm{AUC}}_{2}$ on negative variance rates. In this study, 4 readers read the same 67 diseased and 33 nondiseased cases.

(a) OR estimates for original Franken et al.^22^ data set computed using the unbiased covariance method with empirical AUC as the outcome.									
${\hat{\mathrm{AUC}}}_{1}$	${\hat{\mathrm{AUC}}}_{2}$	${\hat{\sigma }}_{R:\mathrm{OR}}^{2}$	${\hat{\sigma }}_{\tau R:\mathrm{OR}}^{2}$	${\hat{\sigma }}_{\varepsilon :\mathrm{OR}}^{2}$	${\hat{r}}_{1}$	${\hat{r}}_{2}$	${\hat{r}}_{3}$		
0.848	0.837	0.0000433	0.00000	0.00150	0.521	0.320	0.339		
(b) OR parameter values describing simulated data sets.									
${\mathrm{AUC}}_{1}$	${\mathrm{AUC}}_{2}$	$\sigma_{R:\mathrm{OR}}^{2}$	$\sigma_{\tau R:\mathrm{OR}}^{2}$	$\sigma_{\varepsilon :\mathrm{OR}}^{2}$	$r_{1}$	$r_{2}$	$r_{3}$		
0.860	0.820	0.0000433	0.00000	0.00152	0.520	0.335	0.325		
0.840	0.840	0.0000433	0.00000	0.00152	0.510	0.335	0.325		
0.860	0.820	0.0000433	0.00000	0.00152	0.510	0.350	0.310		
0.840	0.840	0.0000433	0.00000	0.00152	0.510	0.350	0.310		
0.860	0.820	0.0000433	0.00020	0.00152	0.510	0.335	0.325		
0.840	0.840	0.0000433	0.00020	0.00152	0.510	0.335	0.325		
0.860	0.820	0.0000433	0.00020	0.00152	0.510	0.350	0.310		
0.840	0.840	0.0000433	0.00020	0.00152	0.510	0.350	0.310		
(c) Parameter values for constrained unequal-variance RM models used to simulate data corresponding to OR parameters in (b).									
$\mu_{1}$	$\mu_{2}$	$\sigma_{R}^{2}$	$\sigma_{\tau R}^{2}$	$\sigma_{C}^{2}$	$\sigma_{TC}^{2}$	$\sigma_{RC}^{2}$	$\sigma_{\varepsilon }^{2}$	b	
2.019568	1.711200	0.001295	0	0.395881	0.006392	0.204401	0.3933260	0.633453	
1.883984	1.883984	0.001312	0	0.392750	0.010942	0.192627	0.4036811	0.621797	
2.019568	1.711200	0.001295	0	0.379211	0.039340	0.211097	0.3703523	0.633453	
1.883984	1.883984	0.001312	0	0.376222	0.043770	0.209155	0.3708535	0.621797	
2.010514	1.703528	0.001283	0.005820	0.396346	0.006403	0.194848	0.4024022	0.638974	
1.874190	1.874190	0.001298	0.005985	0.393143	0.010961	0.193039	0.4028567	0.627792	
2.010514	1.703528	0.001283	0.005820	0.379646	0.039412	0.211548	0.3693941	0.638974	
1.874190	1.874190	0.001298	0.005985	0.376588	0.043846	0.209595	0.3699721	0.627792	
(d) Estimates corresponding to OR parameters in (b) resulting from OR analysis of AUC data simulated using the RM models in (d)									
${\hat{\mathrm{AUC}}}_{1}$	${\hat{\mathrm{AUC}}}_{2}$	${\hat{\sigma }}_{R:\mathrm{OR}}^{2}$	${\hat{\sigma }}_{\tau R:\mathrm{OR}}^{2}$	${\hat{\sigma }}_{\varepsilon :\mathrm{OR}}^{2}$	${\hat{r}}_{1}$	${\hat{r}}_{2}$	${\hat{r}}_{3}$	var < 0 rate	$r_{2}<r_{3}$ rate
0.860	0.820	0.000041688	0.00000	0.00152	0.520	0.334	0.325	2.24%	36%
0.840	0.840	0.000043018	0.00001	0.00152	0.511	0.335	0.325	2.16%	36%
0.860	0.820	0.000046678	0.00000	0.00152	0.509	0.349	0.309	0.31%	8%
0.841	0.840	0.000037359	0.00001	0.00152	0.509	0.349	0.309	0.29%	8%
0.860	0.819	0.000049993	0.00019	0.00152	0.510	0.336	0.326	1.55%	36%
0.840	0.840	0.000049150	0.00020	0.00152	0.510	0.336	0.325	1.48%	36%
0.860	0.820	0.000024317	0.00020	0.00152	0.510	0.350	0.310	0.25%	8%
0.841	0.840	0.000052278	0.00020	0.00152	0.509	0.350	0.310	0.27%	7%

Note: “(var < 0) rate” = proportion of samples where var^OR;unconstrained(θ^1•−θ^2•)<0.

**Fig. 2:**
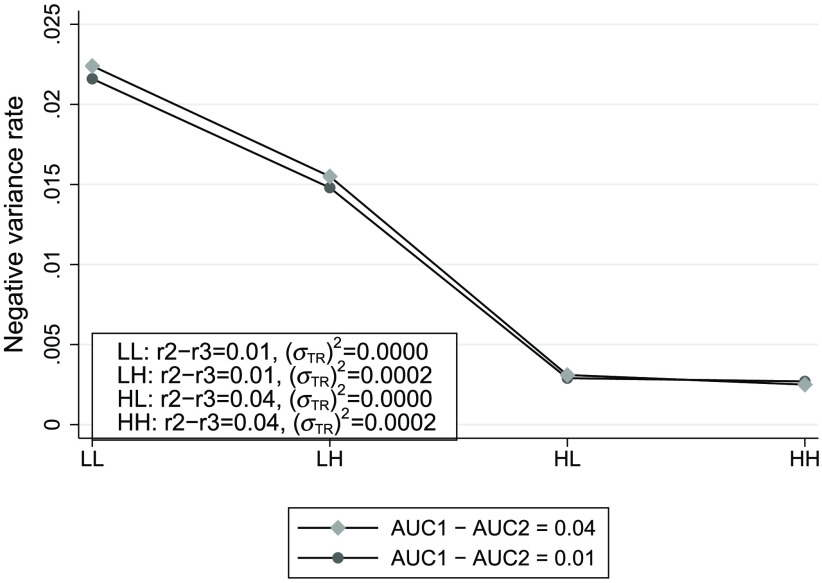
Negative unconstrained OR AUC-difference variance rates, as defined by Eq. (36), for different combinations of OR parameter values. RM model parameter values used for simulating the data are similar to estimates from an OR analysis of the Franken et al.^22^ data. There were 10,000 simulated MRMC samples for each set of RM parameter values, with each sample corresponding to 4 readers reading the same 67 abnormal and 33 normal abdominal radiographs.

Figure 2 displays a plot of the negative variance rate for each combination of the true values of $r_{2}-r_{3},\sigma_{\tau R}^{2}$ and ${\mathrm{AUC}}_{1}-{\mathrm{AUC}}_{2}$. Similar to Fig. 1, higher negative variance rates are associated with lower levels of both $r_{2}-r_{3}$ and $\sigma_{\tau R:\mathrm{OR}}^{2}$, in agreement with the conjectures in the previous section. The effect of ${\mathrm{AUC}}_{1}-{\mathrm{AUC}}_{2}$ is minimal, with the negative variance rate slightly higher for the larger magnitude of ${\mathrm{AUC}}_{1}-{\mathrm{AUC}}_{2}$ when $r_{2}-r_{3}$ is at its low level, as shown by the first two pairs of points.

#### 8.2.4 Summary of simulation results

From the results of the simulations in Secs. 8.2.2 and 8.2.3, we saw that the negative variance problem for the unconstrained OR method is present even when conditions Eqs. (38)–(40), which are implied by the identical-test RM model, do not hold. Moreover, the simulations supported the conjectures given in Sec. 7. Specifically, we saw that while higher negative variance rates were associated with lower levels of both $r_{2}-r_{3}$ and $\sigma_{\tau R:\mathrm{OR}}^{2}$, there was little association with the magnitude of ${\mathrm{AUC}}_{1}-{\mathrm{AUC}}_{2}$. Surprisingly, the largest effect of the magnitude of the AUC difference, shown by the first pair of points in Figs. 1 and 2, shows the negative variance rate to be higher for the larger AUC difference magnitude of 0.04.

In summary, these results suggest that negative variance estimates can be a problem for the unconstrained OR procedure when $r_{2}-r_{3}$ and $\sigma_{\tau R:\mathrm{OR}}^{2}$ are small, regardless of the difference in the AUCs, with the negative variance rate diminishing with increasing numbers of readers and cases. However, we caution that these findings are based on only two simulation studies and will need to be confirmed by additional studies.

## 9 Summary and Discussion

Sometimes it is of interest to compare two tests that may be similar in most respects, such as when noninferiority or equivalence testing is appropriate. For this situation it is important to be able to assess how well a particular MRMC analysis method performs, and hence there is a need for simulation models that emulate this situation. This need was the motivation for developing the RM identical-test model, where the two tests are exactly the same.

The derivation of the RM identical-test model from a particular RM null model was straightforward: simply change the test subscript for all of the RM null model effects to 1, which results in none of the test effects depending on test. This derivation was illustrated for the unconstrained unequal-variance RM model,^9^ which includes the constrained equal-variance^6^ and original^5^ RM null models as special cases. It was shown that the null and corresponding identical-test model rating distributions are the same and the within-test covariances are the same, but the between-test covariances for the null model can be less than those for the identical-test model. In terms of the reader empirical AUCs computed from ratings generated from the identical-test model, it was shown that the expected test empirical AUC estimates are equal, ${\mathrm{Cov}}_{2}$ and ${\mathrm{Cov}}_{3}$ are equal, and $\sigma_{\tau R:\mathrm{OR}}^{2}=0$.

The RM identical-test simulations showed how the performance of the unconstrained OR method is unacceptable because of a nontrivial percentage of negative variance estimates. Because negative estimates can occur, the significance level cannot be estimated unless the action to be taken when a negative estimate occurs has been specified in advance of the analysis and is incorporated into the simulation study. In contrast, the conventional OR method did not have the negative variance problem because its variance estimate can never be negative, and it had an acceptable type I error rate.

The original RM null model^5^ simulations also revealed that the unconstrained OR variance estimate could be negative, but the rates were much smaller than for the identical-test model.

Of course, in practice we would rarely expect two tests to be identical. But if an analysis method does not perform satisfactorily when two tests are exactly the same, then it seems likely that the performance will also not be acceptable when the tests are “close” to being identical. This situation was illustrated in the simulations in Sec. 8, where RM models were created to result in OR distributions somewhat similar to those for two real datasets. In both of those examples, there were nontrivial rates of negative variance estimates (3.2% and 1.55%) with moderate deviations from an identical-test model with respect to two categories (${\mathrm{AUC}}_{1}-{\mathrm{AUC}}_{2}=0.04$ and $\sigma_{\tau R:\mathrm{OR}}^{2}=0.0002)$ and a slight deviation with respect to the other category ($r_{2}-r_{3}=0.01$). Furthermore, the results of the two examples in Sec. 8 suggest that increasing the AUC difference does not reduce the negative-variance rate; if future research shows this relationship to hold in general, then this result implies that negative variance rates can be nontrivial even when the AUC difference is substantial.

Although there has never been any suggestion in the literature that the unconstrained version of OR should be used instead of the conventional version, the findings of this paper are relevant because of the relationship between the unconstrained OR method and the often-used Gallas analysis method. As discussed in Sec. 5, recently^17^ it has been shown that the Gallas test statistic for comparing two tests is equivalent to the unconstrained OR test statistic when empirical AUC is the outcome and the unbiased error-covariance method is used. Thus we recommend that the Gallas method not be used. For the Gallas method to be a statistically acceptable method, there would have to be a defined follow-up analysis procedure to use if the Gallas variance is negative, as well as simulation studies validating the performance of this two-step approach.

In my opinion, it is much easier to interpret an RM model in terms of the OR parameter values describing the resulting empirical AUC distribution based on data simulated from the model, as opposed to interpreting the RM parameter values in terms of the distribution of the confidence-of-disease ratings. It was shown in Sec. 5.5 that an unconstrained unequal-variance RM identical-test model will have an empirical AUC distribution with ${\mathrm{Cov}}_{2}-{\mathrm{Cov}}_{3}=0$ (or equivalently, $r_{2}-r_{3}=0$), no reader-by-test interaction, and equal expected test AUC values. These three OR relationships are intuitively obvious for identical tests and they can be thought of as the criteria by which tests can be identical in terms of the empirical AUC distributions.

In contrast, it has been shown [Ref. 9, Tables 4 and 6] that the original^5^ 12 sets of RM model parameter values lead to OR distributions with identical expected test AUC values, but with $0.05<r_{2}-r_{3}<0.29$, and for 10 of the sets, $0.000287<\sigma_{\tau R:\mathrm{OR}}^{2}<0.001629$; for the other 2 sets, $\sigma_{\tau R:\mathrm{OR}}^{2}=0.00004$. To obtain some perspective on the size of the interaction variance component, we note that $\sigma_{\tau R:\mathrm{OR}}^{2}=0.0002$ implies that the middle 95% probability range is 0.08 for the true ${\mathrm{AUC}}_{1}-{\mathrm{AUC}}_{2}$ difference for a randomly selected reader, as discussed in Hillis and Schartz;^23^ for this reason, we consider $\sigma_{\tau R:\mathrm{OR}}^{2}=0.0002$ to be at least moderate test-by-reader interaction. Thus, in terms of the 3 OR identical-test criteria, 10 of the 12 original RM parameter sets describe tests that are similar with respect only to the OR equal-test AUC criterion, with the other 2 sets describing tests also approximately similar with respect to the OR $\sigma_{\tau R:\mathrm{OR}}^{2}=0$ criterion. But none of them describes tests that are approximately similar with respect to all three OR criteria.

In summary, the RM identical-test model is useful because it allows for assessment of an MRMC analysis method for the situation where the two tests are identical and it is easy to derive from a previously formulated RM null model. Ideally, an MRMC analysis method would be assessed with respect to a wide range underlying rating models. Thus the RM identical-test model should typically be used in conjunction with other RM models.

For brevity, results of the simulation studies in this paper have been limited to the minimum needed to accomplish the two purposes of the paper: to show how to formulate an identical-test RM model and to show its usefulness for validating the need for the OR error covariance constraints. For example, a more extensive analysis might include estimating the type I error, not just for the two-sided nonequivalence set of hypotheses, but also for the noninferiority and equivalence sets of hypotheses; reporting results in Tables 4 and 5 for each structure instead of averaging across the four structures; and reporting results for more combinations of RM parameter values.

Finally, for future research, I recommend creating a new set of RM model parameter sets that correspond to real datasets, as was done in Sec. 8. Doing this will allow for a better understanding of what types of studies are emulated by the simulated data. Recently^21^ an algorithm has been developed that maps OR parameter estimates obtained from real datasets to constrained unequal-variance RM model parameter values; this algorithm can be easily implemented using the R function OR_to_RMH, available in the R package MRMCaov.^19^ This algorithm was utilized in Sec. 8 to create the RM parameter values corresponding to the two real-datasets.

## 10 Appendix A Derivation of the Relationships Between the OR Model and Unconstrained Unequal-Variance RM Identical-Test Model Given in Sec. 5.5

The OR parameters that describe the distribution of the empirical AUC estimates computed from MRMC data simulated from the unconstrained unequal-variance RM model have been expressed as functions of the RM model parameters by Hillis.^9^ Table 7 presents the relationships for the three OR parameters given in Sec. 5.5.

**Table 7:** The OR ${\mathrm{Cov}}_{2}$ and ${\mathrm{Cov}}_{3}$ parameters corresponding to empirical AUC estimates computed from MRMC data simulated from the unconstrained unequal-variance RM model, expressed as functions of the RM model parameters.

$\mu_{\mathrm{OR}}+\tau_{i:\mathrm{OR}}=\Phi (\frac{\delta_{i}}{\sqrt{V}})$
${\mathrm{Cov}}_{2}=\frac{1}{2}\overset{2}{\underset{i=1}{\sum }}\overset{4}{\underset{m=1}{\sum }}c_{m}F_{\mathrm{BVN}}(\frac{\delta_{i}}{\sqrt{V}},\frac{\delta_{i}}{\sqrt{V}};\frac{\rho_{m}(\sigma_{\text{fixed}(-)}^{2}+\sigma_{\text{fixed}(+)}^{2})}{V})$
where $\rho_{1}=\frac{\sigma_{TC(-)}^{2}+\sigma_{C(-)}^{2}+\sigma_{TC(+)}^{2}+\sigma_{C(+)}^{2}}{\sigma_{\text{fixed}(-)}^{2}+\sigma_{\text{fixed}(+)}^{2}},\;\rho_{2}=\frac{\sigma_{TC(-)}^{2}+\sigma_{C(-)}^{2}}{\sigma_{\text{fixed}(-)}^{2}+\sigma_{\text{fixed}(+)}^{2}},\;\rho_{3}=\frac{\sigma_{TC(+)}^{2}+\sigma_{C(+)}^{2}}{\sigma_{\text{fixed}(-)}^{2}+\sigma_{\text{fixed}(+)}^{2}},\;\rho_{4}=0$
${\mathrm{Cov}}_{3}=\overset{4}{\underset{m=1}{\sum }}c_{m}F_{\mathrm{BVN}}(\frac{\delta_{1}}{\sqrt{V}},\frac{\delta_{2}}{\sqrt{V}};\frac{\rho_{m}(\sigma_{\text{fixed}(-)}^{2}+\sigma_{\text{fixed}(+)}^{2})}{V})$
where $\rho_{1}=\frac{\sigma_{C(-)}^{2}+\sigma_{C(+)}^{2}}{\sigma_{\text{fixed}(-)}^{2}+\sigma_{\text{fixed}(+)}^{2}},\;\rho_{2}=\frac{\sigma_{C(-)}^{2}}{\sigma_{\text{fixed}(-)}^{2}+\sigma_{\text{fixed}(+)}^{2}},\;\rho_{3}=\frac{\sigma_{C(+)}^{2}}{\sigma_{\text{fixed}(-)}^{2}+\sigma_{\text{fixed}(+)}^{2}},\;\rho_{4}=0$
$\sigma_{R:\mathrm{OR}}^{2}=F_{\mathrm{BVN}}(\frac{\delta_{1}}{\sqrt{V}},\frac{\delta_{2}}{\sqrt{V}};\frac{2\sigma_{R}^{2}}{V})-[\Phi (\frac{\delta_{1}}{\sqrt{V}})\Phi (\frac{\delta_{2}}{\sqrt{V}})]$
$\sigma_{\tau R:\mathrm{OR}}^{2}=.5\overset{2}{\underset{i=1}{\sum }}\{F_{\mathrm{BVN}}(\frac{\delta_{i}}{\sqrt{V}},\frac{\delta_{i}}{\sqrt{V}};\frac{2(\sigma_{R}^{2}+\sigma_{\tau R}^{2})}{V})-{\left[\Phi (\frac{\delta_{i}}{\sqrt{V}})\right]}^{2}\}-\sigma_{R:\mathrm{OR}}^{2}$

Notes: these results are taken from Table 3 in Hillis.^9^ $F_{\mathrm{BVN}}(.,.;\rho )$ is the standardized bivariate normal distribution function with correlation ρ; $\delta_{i}=\mu_{+}+\tau_{i+}$; $V=\sigma_{\text{fixed}(-)}^{2}+\sigma_{\text{fixed}(+)}^{2}+2(\sigma_{R}^{2}+\sigma_{\tau R}^{2})$, where $\sigma_{\text{fixed}(-)}^{2}=\sigma_{C(-)}^{2}+\sigma_{\tau C(-)}^{2}+\sigma_{RC(-)}^{2}+\sigma_{\varepsilon (-)}^{2}$ and $\sigma_{\text{fixed}(+)}^{2}=\sigma_{C(+)}^{2}+\sigma_{\tau C(+)}^{2}+\sigma_{RC(+)}^{2}+\sigma_{\varepsilon (+)}^{2}$; $c_{1}=1/(n_{0}n_{1})$; $c_{2}=(n_{1}-1)/(n_{0}n_{1})$; $c_{3}=(n_{0}-1)/(n_{0}n_{1})$; and $c_{4}=(1-n_{0}-n_{1})/(n_{0}n_{1})$.

The unconstrained unequal-variance RM model assumed in Table 7 is the same as the unconstrained unequal-variance RM null model discussed in Sec. 5.5, but with the addition of the test-by-truth interaction effect $\tau_{it}$ to the mixed linear model Eq. (1). It follows that the expected difference between the nondiseased and diseased decision-variable distributions is $\delta_{1}=\mu_{\mathrm{pos}}+\tau_{1+}$ for test 1 and $\delta_{2}=\mu_{\mathrm{pos}}+\tau_{2+}$ for test 2.

Relationships Eqs. (38)–(40) in Sec. 5.5 are for the unconstrained unequal-variance RM identical-test model, which is the same as the model assumed in Table 7 with the following constraints imposed

$$\sigma_{\tau R}^{2}=\sigma_{\tau C(+)}^{2}=\sigma_{\tau C(-)}^{2}=\sigma_{\tau RC(+)}^{2}=\sigma_{\tau RC(-)}^{2}=0,$$ (45)

and

$$\delta_{1}=\delta_{2}.$$ (46)

Relationships Eqs. (38)–(40) follow directly from the results in Table 8 when constraints Eqs. (45) and (46) are imposed. Specifically

$${\mathrm{Cov}}_{2}={\mathrm{Cov}}_{3}=\overset{4}{\underset{m=1}{\sum }}c_{m}F_{\mathrm{BVN}}(\frac{\delta_{1}}{\sqrt{V}},\frac{\delta_{1}}{\sqrt{V}};\frac{\rho_{m}(\sigma_{\text{fixed}(-)}^{2}+\sigma_{\text{fixed}(+)}^{2})}{V}),$$

where

$$\delta_{1}=\mu_{+}+\tau_{i+},\;\rho_{1}=\frac{\sigma_{C(-)}^{2}+\sigma_{C(+)}^{2}}{\sigma_{\text{fixed}(-)}^{2}+\sigma_{\text{fixed}(+)}^{2}},\;\rho_{2}=\frac{\sigma_{C(-)}^{2}}{\sigma_{\text{fixed}(-)}^{2}+\sigma_{\text{fixed}(+)}^{2}},\;\rho_{3}=\frac{\sigma_{C(+)}^{2}}{\sigma_{\text{fixed}(-)}^{2}+\sigma_{\text{fixed}(+)}^{2}},\;\rho_{4}=0,$$ (47)

and

$$\mu_{\mathrm{OR}}+\tau_{1;\mathrm{OR}}=\mu_{\mathrm{OR}}+\tau_{2;\mathrm{OR}}=\Phi (\frac{\delta_{1}}{\sqrt{V}}),$$

where

$$V=\sigma_{\text{fixed}(-)}^{2}+\sigma_{\text{fixed}(+)}^{2}+2(\sigma_{R}^{2}+\sigma_{\tau R}^{2})=\sigma_{C(-)}^{2}+\sigma_{RC(-)}^{2}+\sigma_{\varepsilon (-)}^{2}+\sigma_{C(+)}^{2}+\sigma_{RC(+)}^{2}+\sigma_{\varepsilon (+)}^{2}+2(\sigma_{R}^{2}+\sigma_{\tau R}^{2}).$$

**Table 8:** 12 original sets of Roe and Metz^5^ (RM) null simulation model parameter values and corresponding RM identical-test model parameter values. Table 3 is a subset of this table.

Line	Structure	$\mu_{+}$	$A_{z}$	$\sigma_{C}^{2}$	$\sigma_{TC}^{2}$	$\sigma_{RC}^{2}$	$\sigma_{\varepsilon }^{2}$	$\sigma_{R}^{2}$	$\sigma_{\tau R}^{2}$
(a) Original RM null model parameter values									
1	HL	0.75	0.702	0.3	0.3	0.2	0.2	0.0055	0.0055
2	LL	0.75	0.702	0.1	0.1	0.2	0.6	0.0055	0.0055
3	HH	0.75	0.702	0.3	0.3	0.2	0.2	0.0110	0.0110
4	LH	0.75	0.702	0.1	0.1	0.2	0.6	0.0110	0.0110
5	HL	1.5	0.856	0.3	0.3	0.2	0.2	0.0055	0.0055
6	LL	1.5	0.856	0.1	0.1	0.2	0.6	0.0055	0.0055
7	HH	1.5	0.856	0.3	0.3	0.2	0.2	0.0300	0.0300
8	LH	1.5	0.856	0.1	0.1	0.2	0.6	0.0300	0.0300
9	HL	2.5	0.961	0.3	0.3	0.2	0.2	0.0055	0.0055
10	LL	2.5	0.961	0.1	0.1	0.2	0.6	0.0055	0.0055
11	HH	2.5	0.961	0.3	0.3	0.2	0.2	0.0560	0.0560
12	LH	2.5	0.961	0.1	0.1	0.2	0.6	0.0560	0.0560
(b) Corresponding RM identical-test model parameter values									
1	HL	0.75	0.702	0.6	0	0.2	0.2	0.0110	0.0000
2	LL	0.75	0.702	0.2	0	0.2	0.6	0.0110	0.0000
3	HH	0.75	0.702	0.6	0	0.2	0.2	0.0220	0.0000
4	LH	0.75	0.702	0.2	0	0.2	0.6	0.0220	0.0000
5	HL	1.5	0.856	0.6	0	0.2	0.2	0.0110	0.0000
6	LL	1.5	0.856	0.2	0	0.2	0.6	0.0110	0.0000
7	HH	1.5	0.856	0.6	0	0.2	0.2	0.0600	0.0000
8	LH	1.5	0.856	0.2	0	0.2	0.6	0.0600	0.0000
9	HL	2.5	0.961	0.6	0	0.2	0.2	0.0110	0.0000
10	LL	2.5	0.961	0.2	0	0.2	0.6	0.0110	0.0000
11	HH	2.5	0.961	0.6	0	0.2	0.2	0.1120	0.0000
12	LH	2.5	0.961	0.2	0	0.2	0.6	0.1120	0.0000

Notes: $\mu_{+}$ is the median and mean separation of the normal and abnormal DV distributions across the reader population, and $A_{z}=\Phi (\mu_{+}/\sqrt{2})$ is the median reader-specific true area under the ROC curve.

In addition, we can write

$$\sigma_{R:\mathrm{OR}}^{2}=F_{\mathrm{BVN}}(\frac{\delta_{1}}{\sqrt{V}},\frac{\delta_{2}}{\sqrt{V}};\frac{2\sigma_{R}^{2}}{V})-[\Phi (\frac{\delta_{1}}{\sqrt{V}})\Phi (\frac{\delta_{2}}{\sqrt{V}})]\;=F_{\mathrm{BVN}}(\frac{\delta_{1}}{\sqrt{V}},\frac{\delta_{1}}{\sqrt{V}};\frac{2\sigma_{R}^{2}}{V})-[\Phi {\left(\frac{\delta_{1}}{\sqrt{V}}\right)}^{2}],$$

and the first term on the right in the Table 7 expression for $\sigma_{\tau R:\mathrm{OR}}^{2}$ can be expressed in the form

$$5\overset{2}{\underset{i=1}{\sum }}\{F_{\mathrm{BVN}}(\frac{\delta_{i}}{\sqrt{V}},\frac{\delta_{i}}{\sqrt{V}};\frac{2(\sigma_{R}^{2}+\sigma_{\tau R}^{2})}{V})-{\left[\Phi (\frac{\delta_{i}}{\sqrt{V}})\right]}^{2}\}\;=5\overset{2}{\underset{i=1}{\sum }}\{F_{\mathrm{BVN}}(\frac{\delta_{1}}{\sqrt{V}},\frac{\delta_{1}}{\sqrt{V}};\frac{2(\sigma_{R}^{2}+\sigma_{\tau R}^{2})}{V})-{\left[\Phi (\frac{\delta_{1}}{\sqrt{V}})\right]}^{2}\}\;=\{F_{\mathrm{BVN}}(\frac{\delta_{1}}{\sqrt{V}},\frac{\delta_{1}}{\sqrt{V}};\frac{2(\sigma_{R}^{2}+\sigma_{\tau R}^{2})}{V})-{\left[\Phi (\frac{\delta_{1}}{\sqrt{V}})\right]}^{2}\}\;=\sigma_{R:\mathrm{OR}}^{2}(\text{using the previous result}).$$

Replacing the first term on the right in the Table 7 expression for $\sigma_{\tau R;\mathrm{OR}}^{2}$ by $\sigma_{R:\mathrm{OR}}^{2}$ yields

$$\sigma_{\tau R:\mathrm{OR}}^{2}=\sigma_{R:\mathrm{OR}}^{2}-\sigma_{R:\mathrm{OR}}^{2}=0.$$

## 11 Appendix B Original Roe and Metz Null Simulation Model Parameter Values and Corresponding RM Identical-Test Model Parameter Values

For completeness, Table 8 lists the 12 sets of the orginal Roe and Metz^5^ (RM) null simulation model parameter values and the corresponding RM identical-test model parameter values. Table 3 is a subset of this table.

## Code, Data, and Materials Availability

The two datasets (VanDyke and Kundel) analyzed in this article are publicly available as part of the R package MRMCaov.^19^ Code for performing the conventional OR analysis using either the unbiased or DeLong or jackknife error covariance methods is also included in the MRMCaov package. Although code for performing unconstrained OR analysis is not included in MRMCaov, one can perform the unconstrained OR analysis based on the MRMCaov conventional OR analysis output.
